# PKD1 and PKD2 mutations in Slovenian families with autosomal dominant polycystic kidney disease

**DOI:** 10.1186/1471-2350-7-6

**Published:** 2006-01-23

**Authors:** Katja Vouk, Lana Strmecki, Jitka Stekrova, Jana Reiterova, Matjaz Bidovec, Petra Hudler, Anton Kenig, Simona Jereb, Irena Zupanic-Pajnic, Joze Balazic, Guido Haarpaintner, Bostjan Leskovar, Anton Adamlje, Antun Skoflic, Reina Dovc, Radovan Hojs, Radovan Komel

**Affiliations:** 1 Medical Centre for Molecular Biology, Institute of Biochemistry, Faculty of Medicine, Vrazov trg 2, 1000 Ljubljana, Slovenia; 2 Children's Hospital Ljubljana, Clinic for Paediatric Nephrology and Radiology Unit, Vrazov trg 1, 1000 Ljubljana, Slovenia; 3 Institute of Forensic Medicine, Faculty of Medicine, Korytkova 2, 1000 Ljubljana, Slovenia; 4 Trbovlje General Hospital, Dialysis Department, Rudarska 7, Trbovlje, Slovenia; 5 Celje General Hospital, Nephrology Department and Dialysis Centre, Oblakova 5, 3000 Celje, Slovenia; 6 Maribor General Hospital, Clinical Department for Internal Medicine, Nephrology Department, 2000 Maribor, Slovenia; 7 Department of Medical Genetics and Department of Nephrology,1^*st *^Faculty of Medicine, Charles University, Albertov 2, 12800 Prague 2, Czech Republic

## Abstract

**Background:**

Autosomal dominant polycystic kidney disease (ADPKD) is a genetically heterogeneous disorder caused by mutations in at least two different loci. Prior to  performing mutation screening, if DNA samples of  sufficient number of family members are available, it  is worthwhile to assign the gene involved in disease  progression by the genetic linkage analysis.

**Methods:**

We collected samples from 36 Slovene ADPKD families and performed linkage analysis in 16 of them. Linkage was assessed by the use of microsatellite polymorphic markers, four in the case of *PKD1 *(KG8, AC2.5, CW3 and CW2) and five for *PKD2 *(D4S1534, D4S2929, D4S1542, D4S1563 and D4S423). Partial *PKD1 *mutation screening was undertaken by analysing exons 23 and 31–46 and *PKD2 *.

**Results:**

Lod scores indicated linkage to *PKD1 *in six families and to *PKD2 *in two families. One family was linked to none and in seven families linkage to both genes was possible. Partial *PKD1 *mutation screening was performed in 33 patients (including 20 patients from the families where linkage analysis could not be performed). We analysed *PKD2 *in 2 patients where lod scores indicated linkage to *PKD2 *and in 7 families where linkage to both genes was possible. We detected six mutations and eight polymorphisms in *PKD1 *and one mutation and three polymorphisms in *PKD2*.

**Conclusion:**

In our study group of ADPKD patients we detected seven mutations: three frameshift, one missense, two nonsense and one putative splicing mutation. Three have been described previously and 4 are novel. Three newly described framesfift mutations in *PKD1 *seem to be associated with more severe clinical course of ADPKD. Previously described nonsense mutation in PKD2 seems to be associated with cysts in liver and milder clinical course.

## Background

Autosomal dominant polycystic kidney disease (ADPKD; MIM:173900) is a common genetic disease of the kidney, with a population frequency of ~0.1% [1]. It is characterized by progressive renal cystic disease, typically leading to end-stage renal disease (ESRD) in late middle age. ADPKD accounts for approximately 5% of ESRD in western countries. The rate of progression towards kidney failure is variable and whilst end-stage renal failure (ESRF) most commonly occurs in the fifth decade [2] it can present as early as *in utero *whereas some individuals may never progress to renal failure [3-5]. The disorder is geneticaly heterogeneous with two genes implemented; *PKD1 *on 16p13.3 (MIM:601313) and *PKD2 *on 4q21-23 (MIM:173910) [6,7]. Cases of families unlinked to either of the loci have also been described [8]. *PKD1 *accounts for approximately 85% of cases and is associated with a more severe disease course (average age at onset of ESRD of 53 compared to 69 years for *PKD2*) [9]. Renal cysts are likely to develop only after a second-hit or somatic mutation, which inactivates the inherited normal allele of the same locus, or occasionally an allele of another counterpart locus, giving rise to a transheterozygous event [10].

Polycystin-1, the protein encoded by *PKD1*, is predicted to be a large transmembrane glycoprotein [11,12]. *PKD2 *codes for polycystin-2 which functions as a non-selective cation channel that can conduct calcium ions [7,13]. It has been shown that the two proteins interact with each other via their C-terminal regions and possibly function as flow-sensitive mechanosensors in the primary cilium of the renal epithelium cells [14]. A failure of fluid-flow sensation of the cells may disturb tissue morphogenesis and trigger abnormal cell proliferation and cyst formation.

Although presymptomatic diagnosis of ADPKD is possible by various imaging methods and is relatively reliable in adult patients, genetic diagnosis is important for pre-symptomatic diagnosis in younger individuals and in cases with no family history of the disease. Mutation screening in ADPKD is a cumbersome and expensive process as *PKD1 *is large and most of it is reiterated in the form of homologues genes (HG) at least six times on the same chromosome [15]. The presence of HG complicates mutation screening in *PKD1 *as classic PCR amplifies both *PKD1 *as well as HG sequences. Several groups have already successfully screened most of the coding region of *PKD1 *[15-22]. From these studies it is apparent that mutations are dispersed across the entire *PKD1 *and *PKD2 *genes with no clustering in mutation 'hot spots'. The majority of them are found to be private. It is therefore worthwhile to first assign the gene involved in a particular ADPKD family before proceeding with mutation screening. Nevertheless less than half of our ADPKD families fitted to the criteria for performing linkage analysis and only in those families could we preselect the ones suitable for *PKD1 *or *PKD2 *screening. Due to a limited budget we have screened only a part of the *PKD1 *gene and the entire *PKD2 *gene.

## Methods

### Patients and families

We collected samples of 36 Slovene ADPKD families. Linkage studies were performed in 16 families (comprising 48 patients and 41 healthy family members) where samples of at least three affected members or two affected and two unaffected members were available. Ultrasound examination and confirmed history of ADPKD in the families was required to enter the study. Diagnosis of ADPKD was based on ultrasonographic criteria described by Ravine at al., 1994 [23]. The study was approved by the Slovenian Medical Ethics Committee and research was conducted in accordance with the Helsinki Declaration.

### Linkage analysis

Genomic DNA was extracted from white blood cells by the standard salting out procedure [24]. We developed three fluorescent multiplex PCR reactions for simultaneous amplification of 9 polymorphic *PKD1 *and *PKD2 *associated microsatellites: D16S3252 (KG8), D16S665 (SM6), D16S291 (AC2.5), D16S664 (CW3), D16S663 (CW2), D4S1534, D4S2929, D4S1563 and D4S423. We determined amplified product lengths using capillary electrophoresis. Details about primer pairs, labelling, cycling conditions and capillary electrophoresis procedures have been described previously [25].

In addition, we also analysed two intragenic RFLPs, p.A4058V and p.A4091A as described previously [26]. Prior to performing linkage analysis we characterised the number of alleles, their sizes, frequencies and heterozygosity content for each microsatellite in a group of 27–42 unrelated individuals. Then we calculated two-point Lod scores using MLINK and ILINK options of LINKAGE 5.1 [27]. In order to be able to calculate Lod scores in batches for all families and pairs of loci we added two small subprograms to the LINKAGE 5.1 *pedin.bat *accordingly.

A gene frequency of 0.001 was assumed for *PKD1 *and 0.0001 for *PKD2*. For *PKD1*, three liability classes were assumed, corresponding to gene penetrances of 0.64, 0.92, and 0.99 for age groups 0–10, 10–30 and over 30 years respectively [28,29]. For *PKD2*, the liability classes assumed were 0.50, 0.85, and 0.95 for age groups 0–20, 20–30 and over 30 years respectively [30]. Linkage calculations were carried out under an assumption of no difference between the female and male recombination rate and the absence of genetic interference.

### Mutation screening

We screened *PKD1 *exons 23 and 31–46 for mutations using direct sequencing. Primer sequences and PCR conditions are available on request. Prior to sequencing, we purified PCR products through QIAquick columns (Qiagen QIAquick PCR Purification Kit). We analysed sequences on a Perkin Elmer Thermocycler 9600 using Ready Big Dye Terminator Cycle Sequencing Kit according to the protocol of the producer (Applied Biosystems).

Primer sequences and PCR conditions for *PKD2 *exon amplification are available on request. We performed heteroduplex analysis (HA) using Hydrolink Mutation Detection Enhancement (MDE, BMI) gel solution. Complete PCR products (50 μl) were mixed with 0.5 μl 0.5 M EDTA, denatured at 95°C for 5 min and finally cooled at 37°C for at least 1 hour. We analysed 5:1 ratio of sample and loading dye by electrophoresis through 25 cm-1 × MDE gel with 15% urea at 250 V for 16–24 h. Then we stained gels with ethidium bromide and photographed them under UV light. DNA samples exhibiting shifted bands we amplified and sequenced in both directions using ABI Prism™ 310 Genetic Analyzer (Applied Biosystems) according to the manufacturer's instructions. The detected changes we tested for segregation in each family by either HA or direct sequencing.

### Data analysis

Putative missense changes in *PKD1 *we further analysed for their site conservation in mouse (GenBank:U70209), rat (GenBank:AF277452), cat (GenBank:AF483210) and puffer fish (GenBank:AF013614) cDNA sequences, using BLAST [31]. The effect of these mutations on the predicted secondary structure of the protein was analysed by PHDsec [32,33].

## Results

### Linkage analysis

We calculated linkage to *PKD1 *by only four out of the five microsatellite markers used (/PKD1/-KG8-AC2.5-CW3-CW2). We excluded SM6 from Lod score calculations as the correct determination of alleles was impossible due to stutter products and appearance of new alleles during pedigree analysis (also reported by Peral et al., 1994 [29]). In addition, we used two intragenic RFLPs, p.A4058V and p.A4091A, which helped in deducing allele segregation in some of the families that were not very informative for KG8. Subsequently we did not include them in calculation of Lod scores due to their low informativity. Linkage to *PKD2 *we analysed using five microsatellite markers (D4S1534-D4S2929-D4S1542-/PKD2/-D4S1563-D4S423).

**Figure 1:**
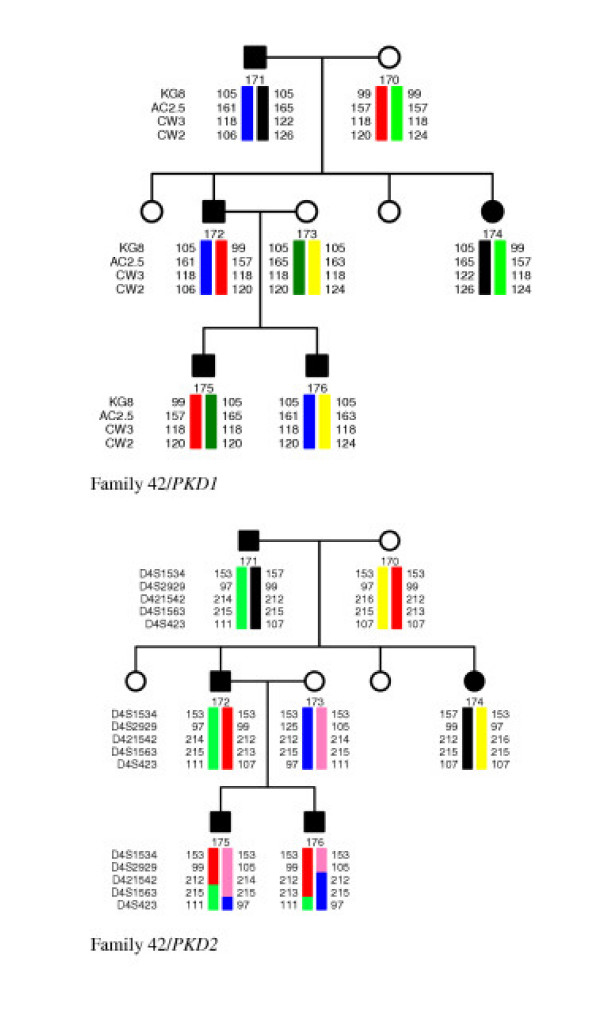
**Pedigree of the family 42 and possible haplotypes for the *PKD1 *(top) and *PKD2 *(bottom) associated polymorphic markers**. Black boxes or circles indicate the affected and empty ones the healthy family members. Numbers indicate lengths of PCR products (in bp) for different alleles of the chosen microsatellites. Names of the *PKD1 *and *PKD2 *associated polymorphic markers are located on the left of the haplotype bars. Changes in colour of the haplotype bars indicate possible recombination events. Lod scores and corresponding maximal recombination fractions (Z_max_, Θ_max_) were calculated for KG8 (-0.86919, 0.035), AC2.5 (-1.738298, 0.035), CW3 (-0.869292, 0.035) and CW2 (-1.738492, 0.035) polymorphic markers. Negative values Z_max _indicate exclusion of linkage to *PKD1*. KG8 lies within 3'-part of the *PKD1 *gene and the other markers are located proximally to the *PKD1 *on 16p13.3.

In six families Lod scores and segregation of alleles indicated probable linkage to *PKD1 *(pedigrees 05, 11, 21, 24, 25 and 36) and in two families to *PKD2 *(pedigrees 10 and 31). Linkage to both genes was possible in seven mostly small families, where the co-segregation is likely to be due to chance (pedigrees 02, 12, 14, 18, 27, 47 and 49). In one of the 16 families analysed (pedigree 42), Lod scores were mostly negative and linkage to neither of the two *PKD *loci could be assumed. [see Figure 1] From segregation of alleles we concluded exclusion of linkage to both *PKD *loci in this pedigree. Table 1 summarises clinical data for the patients of the family 42. All families indicating linkage to *PKD1 *to *PKD2 *or to either *PKD1 *or *PKD2 *were selected for further mutation screening.

**Table 1:** Clinical data of the patients from the family 42

*Patient*	*171/fam 42*	*172/fam 42*	*174/fam 42*	*175/fam42*	*176/fam42*
Gender	M	M	F	M	M
Age (years)	66	38	32	16	14
Age of onset (years)	66	35	32	14	7
Blood pressure	H	H; on therapy	N	N	N
Serum creatinin (μmol/l)	not measured	not measured	80	105	75
Ultrasound of liver	not done	numerous cysts	normal	normal	normal
Ultrasound of kidneys	enlarged kidneys, large cysts	enlarged kidneys, large cysts, calcinations	cysts	several small cysts	several small cysts
Dialysis (age at start in years)	no	no; first signs of insufficiency	no	no	no
Additional symptoms		severe arterial hypertension	headaches		

M or F designate male or female patients. Normal values for blood pressure are less then 120/80 mm Hg, (in the table designated as N) values from 120/80 mm Hg up to 139/89 mm Hg indicate prehypertension (P) and values above 140/90 indicate hypertension (H). Normal values for serum creatinin are 70–115 μmoles/l for men and 52–97 μmoles/l for women.

### Mutation screening

Altogether 33 patients were included in the *PKD1 *mutation screening. We selected one patient from each family where calculated Lod scores did not indicate exclusion of linkage to *PKD1 *(6 families with indicated linkage to *PKD1 *and 7 families where linkage to both genes was possible) and 20 patients from families where linkage analysis was not possible due to lack of family member samples. We screened the *PKD2 *gene for mutations on 9 patients from families where linkage to the *PKD2 *gene could not be excluded (2 families with linkage to *PKD2 *and 7 families where linkage to both genes was possible). We identified seven likely ADPKD causing changes and eleven polymorphisms in both genes. They are summarised in Table 2. Then we checked for segregation of the putative mutation with the ADPKD status, where DNA samples of other family members were available. Table 3 summarises clinical data of the patients from the families where pathogenic mutation was detected.

**Table 2:** Sequence changes identified in PKD1 and PKD2. PKD1 cDNA reference sequence is Entrez:NM_000296 (RefSeq database) and PKD2 cDNA reference sequence is Entrez:NM_000297 (RefSeq database).

Sequence change	Location	Amino Acid Change	Predicted effect on protein	Found in family	Reference
c.8509C>T	exon 23 (*PKD1*)	no change	silent polymorphism	02	present study
c.8522G>A	exon 23 (*PKD1*)	p.E2771K	missense mutation	25	[18]
c.8675G>A	exon 23 (*PKD1*)	p.V2822M	probably polymorphism	09	present study
c.11477+128C>T	intron 39 (*PKD1*)	intronic	polymorphism	19, 25, 39, 43, 46	present study
c.11693_11697dup	exon 41 (*PKD1*)	frameshift	premature stop codon	41	present study
c.11745+3_5dup	intron 41 (*PKD1*)	intronic	possibly influences splicing	38	[34]
c.11820_11845del	exon 42 (*PKD1*)	frameshift	premature stop codon	20	present study
c.12124C>T	exon 43 (*PKD1*)	no change	silent polymorphism	12, 27	present study
c.12341A>G	exon 44 (*PKD1*)	p.I4044V	polymorphism	11, 19, 21, 25, 27, 39, 43, 46, 49	[35]
c.12346+22del	intron 44 (*PKD1*)	intronic	polymorphism	12, 27	[36]
c.12375G>A	exon 45 (*PKD1*)	p.W4055X	nonsense mutation	50	present study
c.12772dup	exon 46 (*PKD1*)	frameshift	premature stop codon	11	present study
c.12838T>C	exon 46 (*PKD1*)	no change	silent polymorphism	11, 19, 21, 25, 27, 39, 43, 46, 49	[37]
c.12973C>T	exon 46 (*PKD1*)	no change	silent polymorphism	20	[38]
c.83G>C	exon 1 (*PKD2*)	p.R28P	polymorphism	31	[39]
c.362C>G	exon 1 (*PKD2*)	p.G121A	probably polymorphism	31	present study
c.844-22 G>A	intron 3 (*PKD2*)	no change	polymorphism	31	[40]
c.916C>T	exon 4 (*PKD2*)	p.R306X	nonsense mutation	10	[40]

**Table 3:** Clinical data of the patients from families 10, 11, 20, 25, 38 and 41 in which we identified presumably pathogenic changes

*Patient*	Mutation/gene	Gender	Age (years)	Age of onset (years)	Blood pressure	Serum creatinin (μmol/l)	Ultrasound of liver	Ultrasound of kidneys	Dialysis (age at start in years)	Additional symptoms
*33 * *fam 10*	c.916C>T p.R306X*/PKD2*	M	51	42	H	147	cysts in the left lobe and gallbladder	enlarged kidneys, large cysts, calcinations	no	cardiac hypertrophy, malfunction of mitral valve
*35 * *fam 10*	c.916C>T p.R306X*/PKD2*	F	23	14	N	62	lesion, arterial angioma	small cysts	no	focal nodular hyperplasia in liver
*36 * *fam 10*	c.916C>T p.R306X*/PKD2*	F	18	9	N	53	normal	small cysts	no	
*37 * *fam 11*	c.12772dup*/PKD1*	M	58+	34	H	1418	cysts, cirrhosis	enlarged kidneys, numerous cysts	yes (46)	kidney stone, obstructive icterus
*40 * *fam 11*	c.12772dup*/PKD1*	F	23	11	N	82	normal	small cysts	no	
*77 * *fam 20*	c.11820_ 11845del*/PKD1*	F	56	46	P	450	cysts	numerous cysts	yes (48)	idiopathic dilated cardiomyopathy
*161 * *fam 20*	c.11820_11845del*/PKD1*	M	47	40	H	399	normal	numerous cysts	yes (44)	persistent hepatitis B
*89 * *fam 25*	c.8522G>A p.E2771K*/PKD1*	M	65	38	N	200	enlarged	numerous cysts	no; first signs of insufficiency	
*90 * *fam 25*	c.8522G>A p.E2771K*/PKD1*	F	42	28	N	normal	enlarged	numerous cysts	no	
*91 * *fam 25*	c.8522G>A p.E2771K*/PKD1*	F	61	40	N	700–800	enlarged with portal hypertension	numerous cysts	yes (43)	headaches
*92 * *fam 25*	c.8522G>A p.E2771K*/PKD1*	M	40	32	N	normal	enlarged	numerous cysts	no	
*166 * *fam 38*	c.11745+3_5 dup*/PKD1*	F	20	9	P	not measured	normal	enlarged kidneys, numerous small cysts	no	endometriosis
*148 * *fam 41*	c.11693_11697dup*/PKD1*	F	44	21	P	458	normal	enlarged kidneys, cysts	yes (44)	gallstones

**Figure 2:**
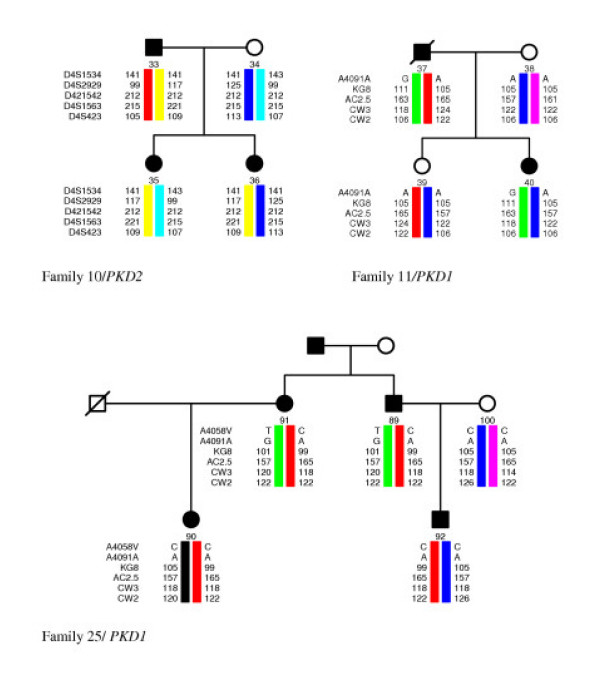
**Pedigrees of the families 10 and 25 with possible haplotypes for the *PKD1 *associated polymorphic markers and pedigree of family 11 with possible haplotypes for the *PKD2 *associated polymorphic markers**. Black boxes or circles indicate the affected and empty ones the healthy family members. Crossed over boxes or circles designate deceased family members. Numbers indicate lengths of PCR products (in bp) for different alleles of the chosen microsatellites. Names of the *PKD1 *and *PKD2 *associated polymorphic markers are located on the left of the haplotype bars.

M or F designate male or female patients. Normal values for blood pressure are less then 120/80 mm Hg, (in the table designated as N) values from 120/80 mm Hg up to 139/89 mm Hg indicate prehypertension (P) and values above 140/90 indicate hypertension (H). Normal values for serum creatinin are 70–115 μmoles/l for men and 52–97 μmoles/l for women. Cross (+) designates death of the patient at the age of 58 years. For relation of the patients in the families 10, 11 and 25 see Figure 2 [see Figure 2]. Patients 77 and 161 from the family 20 are sister and brother. Clinical data for the patient 198 from the family 50 were not complete therefore we did not include them in the Table 3.

We found 3 changes in exon 23 of *PKD1*. The first change is a silent polymorphism c.8509C>T that was detected in family 02. The second change p.E2771K c.8522G>A is a missense mutation already identified in 4 British families [18]. The mutation changes a glutamate residue to a lysine causing a net charge change of the predicted protein. Using the PHDsec computer prediction analysis tool [32,33] we predicted the change to affect the secondary structure of up to 40 amino acid residues upstream. Segregation with the disease was confirmed in the family. Table 3 summarises the clinical data of the four patients and Figure 2. shows a pedigree of the family 25 [see Figure 2]. All four affected relatives have enlarged livers whilst blood pressure remains within normal values.

The third change in exon 23 (*PKD1*) we detected is the substitution from valine to methionine c.8675G>A p.V2822M. We could not analyse for segregation because no additional samples from other family members were available. The valine residue is only conserved in the cDNA sequences of the species cat, mouse and rat, but not puffer fish. Our secondary structure analysis with PHDsec [32,33] shows no drastic effect on the protein structure of this helical domain.

In patient 148 of family 41 we found the duplication of five base pairs in exon 41 (*PKD1*) c.11693_11697dup. The predicted consequence of this duplication is most probably termination of protein synthesis 116 amino acid residues after glutamate 3828. Patient 148 has gallstones and she started dialysis treatment at the age of 44 (rapid progressor). Patient's daughter has enlarged kidneys with multiple cysts at the age of 23 and she suffers from hypertension and proteinuria. Blood sample of the patient's daughter was not avaliable therefore we could not confirm segregation of the mutation in the family.

In family 38 we found the duplication of 3 base pairs in intron 41 (*PKD1*) c.11745+3_5dup. The mutation was described previously in a French patient under different name 11745+2ins3 [34]. We adapted the name of the mutation to the updated recommendation of the Human Genome Variation Society HGVS (May, 2005). The mutation possibly influences splicing of intron 41. We could not analyse segregation due to lack of samples from the family 38.

In two siblings (patients 77 and 161) from family 20 we identified the deletion of 26 base pairs in exon 42 (*PKD1*) c.11820_11845del. The mutation most probably results in protein termination after 80 amino acid residues. Both patients have already rather early started with dialysis treatment: patient 77 at the age of 48 and patient 161 at the age of 44. In patient 77 cysts in the liver and idiopathic dilated cardiomyopathy were detected, while sibling 161 has persistent hepatitis B.

Previously described polymorphism in exon 44 (*PKD1*) c.12341A>G p.I4044V [35] we found in nine apparently unrelated pedigrees in the present study (families 11, 19, 21, 25, 27, 39, 43, 46, 49). In all these families polymorphism c.12341A>G p.I4044V co-segregated with another also previously described silent polymorphism in exon 46 (*PKD1*) c.12838T>C [37].

In two apparently unrelated pedigrees (families 12 and 27) we found previously described intronic deletion in intron 44 (*PKD1*) c.12346+22del [36]. In both families the change does not segregate with the disease nevertheless it co-segregates with a silent polymorphism in exon 43 (*PKD1*) c.12124C>T.

In patient 198 (family 50) we found nonsense mutation in exon 45 (*PKD1 *c.12375G>A p.W4055X. We are lacking clinical data for the patient 198 nevertheless ultrasound was performed for the patient's daughter. She has small cysts in the kidneys that were detected at the age of 5 years. Mother of the patient 198 has a transplanted kidney. Blood samples of the two relatives were not available therefore we could not confirm segregation of the mutation in the family.

In family 11 [see Figure 2] we found the 1 base pair duplication in exon 46 of *PKD1 *c.12772dup. The mutation most probably results in premature protein termination after 20 amino acid residues. The duplication segregates with the disease in the family 11. Patient 37 died at the age of 58 years. He was operated on kidney stones and suffered from liver cirrhosis as well as obstructive icterus. His daughter (23 years) has small cysts in kidneys that were detected at the age of 11.

In *PKD2 *gene we found four changes. In family 10 [see Figure 2] we detected a nonsense mutation in exon 4 *(PKD2*) c.916 C>T p.R306X. The mutation was already described in three Bulgarian families [40] and in two Czech families with a mild clinical course of the disease [41,42]. We confirmed segregation of the mutation with the disease in the family 10. Patient 35 (age 51 years) lives without dialysis treatment. He has enlarged kidneys containing numerous large cysts. He suffers from cardiac hypertrophy as well as from a malfunction of the mitral valve. Focal nodular hyperplasia was detected in the liver of his older daughter (patient 35).

In family 31 we detected three changes in the *PKD2 *gene. In exon 1 (*PKD2*) we found the sequence change c.362 C>G p.G121A. This variation substitutes glycine for alanine at the N-terminal region of polycystin 2. The sequence change however does not segregate with the disease in the family 31. In the same family we also observed the presence of two already described polymorphisms c.83G>C, p.R28P in exon 1 and c.844-22 G>A in intron 3 [39,40].

## Discussion

As both genes associated with ADPKD are large and mutation screening is time consuming and costly, it is prudent to assess linkage to either *PKD1 *or *PKD2 *prior to performing mutation analysis in ADPKD families. Of the 16 families included in linkage analysis, Lod scores and segregation of alleles indicated probable linkage to *PKD1 *in six families (four two-generation families; and in two three-generation families; pedigrees 05, 11, 21, 24, 25 and 36) and linkage to *PKD2 *was indicated in two families (two two-generation families; pedigrees 10 and 31). Linkage to both genes was possible in seven mostly small families, where co-segregation is likely to be due to chance (pedigrees 02, 12, 14, 18, 27, 47 and 49). For one out of the 16 families analysed (pedigree 42), Lod scores were mostly negative and linkage to neither of the two *PKD *loci could be assumed.

We consequently performed mutation screening in either *PKD1 *or *PKD2 *in patients from families with indicative linkage to either one or the other gene and in patients from families where linkage to both genes was possible. In addition we included patients from families where we could not perform linkage studies due to a lack of samples from family members. In total we screened 33 patients for *PKD1 *mutations and 9 patients for *PKD2 *mutations.

Detection of mutations in the duplicated part of *PKD1 *has been hindered by the high (>95%) sequence identity with homologous genes, raising concerns about PCR primer specificity and hence reliability of the analysis [43]. We have focused on a limited area of the gene. In the present study we screened the unduplicated region of *PKD1 *(exons 33–46) as well as exons 31–32 and exon 23 of the duplicated part. Exon 23 was analysed due to its' proximity to the IVS21 polypyrimidine tract, where clustering of mutations has been proposed [44] due to formation of putative triplex DNA structures [45,46]. Although no apparent mutation 'hot spots' were found in *PKD1*, the region around the polypyrimidine tract shows a higher frequency of changes compared to other parts of the gene [18].

Six mutations and eight polymorphisms were detected in *PKD1 *and one mutation and three polymorphisms were detected in *PKD2*. Three mutations were described previously; p.E2771K c.8522G>A (*PKD1*) in four British families [18], c.11745+3_5dup (*PKD1*) in a French patient [34] and p.R306X c.916C>T (*PKD2*) in three Bulgarian [40] and two Czech families [41,42]. Four out of 7 mutations are novel and probably private. All four of them (c.11693_11697dup, c.11820_11845del, c.12375G>A p.W4055X and c.12772dup in *PKD1*) most probably result in premature protein termination.

As there is no functional assay for ADPKD and analysing *PKD1 *in a sufficient number of control chromosomes is costly, the distinction between disease causing mutations and neutral variants has to rely on family segregation studies. Segregation in family we confirmed for four out of seven identified mutations where samples at least two patients from the family were available. From the other three, one mutation was described previously (c.11745+3_5dup) [34], and from the other two c.11693_11697dup is a frameshift and c.12375G>A p.W4055X is a nonsense mutation.

On the basis of collected clinical data of the patients which is summarised in Table 3. we tried to assess the influence of the identified mutations on progression of the disease. Three newly described framesfift mutations in *PKD1 *c.12772dup, c.11820_11845del and c.11693_11697dup seem to be associated with the more severe clinical course of the disease resulting in ESRD (End Stage Renal Disease) in the range from 44 to 48 years (Table 3). Missense mutation in *PKD1 *c.8522G>A p.E2771K seems to be associated either with milder or more severe clinical course in the family 25. Patient 91 reached ESRD early at the age of 43 while patient 89 is showing first signs of insufficiency at the age of 65. Nonsense mutation in *PKD2 *c.916C>T p.R306X seems to be connected to the milder clinical course (slow progress towards ESRD) as already reported by Bulgarian and Czech studies [40-42]. Comparisson to the clinical data of the three Czech patients with age range of 51–81 years (data not shown in the Table 3) reveiled that mutation seems to be associated with cysts in liver.

## Conclusion

In our study group of ADPKD patients six mutations and eight polymorphisms were detected in *PKD1 *and one mutation and three polymorphisms were detected in *PKD2*. Three mutations have been described previously in British, French, Czech and Bulgarian populations. Four out of 7 mutations are novel and probably private. Three newly described framesfift mutations in *PKD1 *seem to be associated with more severe clinical course of the disease resulting in ESRD (End Stage Renal Disease) in the age range of 44 to 48 years. Nonsense mutation in *PKD2 *seems to be associated with cysts in liver and slower progression towards ESRD.

## Competing interests

The author(s) declare that they have no competing interest.

## Authors' contributions

KV carried out genotyping and linkage analysis, majority of mutation screening in PKD1, coordinated experimental work and drafted the manuscript. LS carried out part of mutation screening in PKD1 and contributed to the writing of the manuscript. JS and JR performed mutation screening in PKD2 and contributed to the writing of the manuscript. MB, TK, SJ, BL, AA, AS, RD and RH are nephrologists who were responsible for clinical examinations, contacting the family members of the patients, collecting of clinical data and blood samples. PH took part in PKD1 mutation analysis. IZP and JB carried out paternity testing in one of the families. GH wrote two subprograms that helped us to analyse linkage data. RK supervised the research activity, contributed in study design and revised the manuscript. All the authors read and approved the final manuscript.

## Pre-publication history

The pre-publication history for this paper can be accessed here:


